# Early X-ray workers: an effort to assess their numbers, risk, and most common (skin) affliction

**DOI:** 10.1007/s13244-015-0457-2

**Published:** 2015-12-29

**Authors:** Gerrit J. Kemerink, Jos M. A. van Engelshoven, Kees J. Simon, Gerhard Kütterer, Joachim E. Wildberger

**Affiliations:** Departments of Radiology and Nuclear Medicine, Maastricht University Medical Center, P. Debijelaan 25, 6229 HX Maastricht, The Netherlands

**Keywords:** X-rays, Radiation effects, Skin neoplasms, History nineteenth century, History twentieth century

## Abstract

**Objective:**

To assess quantitatively the number of early X-ray workers, their risk of becoming a radiation victim, and their most common radiation-induced (skin) disease.

**Methods:**

Information on professional life and occupational disease was retrieved from the Ehrenbuch, a book of honour containing biographies of 404 radiation victims, as well as member and congress lists of the German and US radiological societies, obituaries, books, articles, and the Internet.

**Results:**

The estimated numbers of X-ray users in a medical setting in the US increased from about 300 to 600 in 1900–1903, in Germany from about 700 to 1200 during 1905–1908. The risk for a beginning user eventually to die from radiation was 1–2 % in these years, but up to 10–25 % in 1896. Data on 198 victims of fatal radiation-induced skin disease were collected. The incidence of the various stages of skin afflictions with a fatal outcome was characterized by very wide distributions.

**Conclusions:**

After 1896, the radiation risk decreased very fast at first and more slowly thereafter to nearly zero in 1935. Many victims became quite old, partly because of the slower progress of tissue reactions at lower radiation doses, partly because of the success of often multiple surgical interventions.

**Main messages:**

- *US and German X-ray users amounted to several hundreds to thousand in 1900–1908.*
- *The risk eventually to die from radiation was about 1–2 % during 1900–1908.*
- *After 1896, this risk decreased from >10 % to nearly zero in 1935.*
- *The incidence of subsequent stages of skin harm varied strongly in time.*
- *X-ray victims could become quite old, dependent on radiation dose and surgery.*

**Electronic supplementary material:**

The online version of this article (doi:10.1007/s13244-015-0457-2) contains supplementary material, which is available to authorized users.

## Introduction

This year it is 120 years ago that Röntgen discovered his “X-rays” [1]. Within the tradition of keeping the history of the development of radiology alive, we chose to address some aspects relating to early X-ray workers in a quantitative way.

Topics to be looked at are characteristics of the persons who used X-rays, experimental conditions under which they worked, radiation-induced skin afflictions including their temporal development, the number of early X-ray workers in the US and Germany, and their risk of becoming a radiation victim.

Sources consulted were the “Ehrenbuch der Radiologen aller Nationen” [2], early member lists of the US and German X-ray societies [3, 4], lists of visitors of congresses of the German X-ray society [5], obituaries, books [6–15], articles, and the Internet, e.g. newspaper, hospital, and society sites. The Ehrenbuch is a book of honour commemorating all persons, so-called martyrs, who died as a consequence of taking part in the advancement of this new practice. It contains the biographies of 404 victims. A very brief and rather different analysis of X-ray martyrs has been given before by Kerley in 1961 for 160 victims [16].

## Who were the X-ray “martyrs”?

The parameters we extracted to characterise X-ray martyrs were: nationality, year of birth and death, gender, profession, first year of working with X-rays, use of radium and starting year of its use, working with a manufacturing or sales company of equipment or radium, year of first occurrence of chronic dermatitis and its location, year of diagnosis of first malignancy, year of loss of (part) of finger(s) including anatomical location, i.e. left/right/unknown, and similarly for the hand, the arm, and axillary involvement, and finally the cause of death, i.e. metastatic skin cancer, hematologic disease, i.e. leukaemia or (aplastic) anaemia, wasted by radiation, but not obviously radiation-related death, electrocution, and “unknown”. Radium is ^226^Ra, with a half-life of 1600 years, used for therapy.

For all parameters of interest, sufficient sources could be found, but the sets of sources for the various parameters differed generally, as the available information varied per source and parameter. Often the number of sources was, therefore, different as well; in many cases this number will be given as an index of statistical solidity, e.g. as n = 198. In the analysis we included eight Dutch X-ray martyrs [17] who are missing from the Ehrenbuch. Table 1 presents the number of victims (total 412) reported for the various countries. At least two other countries with a victim have recently been reported, and for some of the countries mentioned in Table 1, a few additional martyrs do exist [18].

**Table 1:** Number of victims per country reported in Ehrenbuch + the Netherlands

Australia	4	Finland	2	Israel	2	Russia	13
Austria	10	France	65	Italy	29	Spain	4
Belgium	5	Germany	71	Japan	52	Sweden	1
Czechoslovakia	17	Great Britain	42	Netherlands	8	Switzerland	7
Denmark	5	Greece	1	Poland	1	USA	58
Dutch E. Indies	1	Hungary	11	Portugal	2	Yugoslavia	1

Germany, the “Kaiserreich” of Emperor Wilhelm II at the time of interest, reported the most victims. It was then leading in radiology, with many manufacturers of X-ray equipment [19]. Next in number of victims are France, the USA, Japan, and Great Britain.

The original professions of the victims and their number are given in Table 2.

**Table 2:** Professions of victims in the Ehrenbuch + the Netherlands. Profession was reported for 392 persons of 412 total

Medical doctor	253	Physicist	10	Dentist	2
Technician	57	Mechanic	8	Photographer	1
Nun	5	Chemist	7	Teacher	1
Nurse	6	Chemical assistant	1	Priest	1
Engineer	38	Glass blower	2	Unknown	20

Only 21 women were counted in the whole set of 391 victims with known gender: three medical doctors, two physicists, one chemist, 10 nuns/nurses, four technicians, and one chemical assistant. Among the set of all victims, medical doctors dominate (65 % of those with known profession). The long list of other professions illustrates the variation in background of persons who entered the then new practice. Table 3 shows the reported cause of death of the victims.

**Table 3:** Cause of death of radiation victims (n = 412). Within brackets percentage of all cases with known cause of death (n = 380)

Skin cancer	Hematologic disease	Wasted by radiation	Acute death	Electrocution	Not given
270 (71.1 %)	77 (20.3 %)	23 (6.1 %)	1 (0.3 %)	9 (2.4 %)	32

With a view to further analysis, we notice that the persons in Table 2 fall into three main categories: 1) medical doctors (MD), 2) technicians (tech), and 3) a group that is involved in supplying X-ray systems and radium (sup). This group of “suppliers” includes manufacturers, engineers, physicists, chemists, mechanics, and salesmen owning or working for firms. The rest of the professions (mainly technicians, nuns, and nurses) were considered technicians, with the exception of a small residual group of dentists, physicists and engineers not working for private enterprise, who were added to the group of medical doctors.

**Fig. 1:**
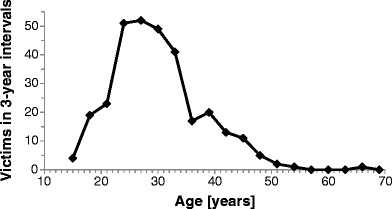
Age at which future victims started using radiation (n = 309)

Figure 1 shows the age of future martyrs when they started radiation work. Realizing most victims were medical doctors who finished training in their mid-20s, the distribution illustrates that many workers began a career in radiology early in their professional live.

Table 4 shows some characteristics for the countries with the most victims. Note that 50 % of all victims in Great Britain and the US started before 1900; worldwide 50 % started before or in 1903. Japan appears to have embraced radiology relatively late, at least on a large scale: about 13 years after Europe and the US. Descriptions of the historical development of radiology in Japan seem to confirm this [20–22].

**Table 4:** Characteristics of X-ray victims in the larger countries. n is number of victims; sd_m is standard deviation of the mean

	Start of radiation work (years)				Age at death (years)			
	n	Average	sd_m	Median	n	Average	sd_m	Median
France	48	1902.4	0.9	1902.0	58	55.6	1.6	55.5
Germany	57	1905.5	1.3	1904.0	63	62.8	1.6	63.0
Great Britain	40	1901.6	1.4	1898.0	40	60.9	2.1	62.0
Japan	47	1920.0	1.3	1919.0	52	60.4	1.7	61.0
USA	41	1901.0	1.3	1897.0	53	56.9	1.9	54.0
All countries	317	1907.2	0.6	1903.0	372	58.7	0.7	59.5

To get an impression of the years of life a radiation victim lost, we calculated a hypothetic lifespan from the age at which a future victim started his radiation work by adding a cohort-based life expectancy at that age and year Anno Domini, using tables for the civil population and distinguishing between male and female persons. The difference between this projected lifespan and the actual years lived by the victim may serve as a measure of years of life lost. We found suitable life expectancy data for France, Germany, the UK, and the USA. The hypothetical lifespan was on average 69.1 years, the actual lifespan was 59.6 years, and the difference was 9.5 years (n = 182). In 43 cases the victim lived longer than the projected lifespan. Details of these calculations are given in Appendix 1. Although the martyrs generally suffered severely, it appears that their loss of years lived was not excessive.

## Estimation of the number of early X-ray users in Germany and the US

Serwer, who prepared a comprehensive thesis on radiology before 1935, wrote [19]: “I have unfortunately been unable to find any contemporary data on the number of X-ray practitioners, but I would guess that by 1900 there were at least several hundred diagnostic X-ray installations in each of the countries of primary concern, namely Austria, England, France, Germany, and the United States.”

**Fig. 2:**
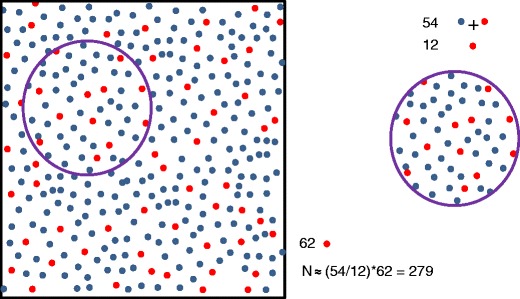
Assessment of number of individuals in a set not accessible for counting. Left (square), the whole population, including a known number of marked individuals (red). Right, a sample from the mother population

Numerical estimates might be obtained, though, by applying the Lincoln-Petersen model [23] to lists of members or congress visitors of professional societies and the names in the Ehrenbuch. The Lincoln-Petersen model is also known as the “mark-recapture model” and it has been applied in widely different contexts. The principle is illustrated in Fig. 2, where all dots in the square represent a group of pioneer radiologists, the number of which we want to know, e.g. in some country. The red dots in the square represent all persons in the Ehrenbuch who were working at the time of assessment; these persons we know by name (equivalent to marked). A list of members or congress visitors can be considered as a selection (“sample”) of the total population (the dots within the circle). After determining the number of working persons from the Ehrenbuch (N_E_), the corresponding number in the sample (N_SE_), and the total number of persons in the sample (N_ST_), one can estimate the size of the total population as N = (N_E_/N_SE_)*N_ST_. This so-called Lincoln-Petersen estimate rests on the assumption that the fraction of markers is identical in the sample (circle) and the mother collection (square). Whether the Ehrenbuch is incomplete or contains persons inappropriately is irrelevant to the method. It is only relevant that it provides a distinguishable set that is part of the population studied. For low “capture rates”, i.e. small N_E_ and N_SE_ compared to N, which we have, one generally uses Chapman’s modification of the model that minimizes bias in the population estimate, but has the same condition for applicability otherwise [24]. For large numbers both models give identical results, otherwise Chapman’s values are slightly lower.

We start considering the earliest lists of members and congress visitors from the “Deutsche Röntgen Gesellschaft” (DRG) as samples taken out of the population of X-ray users in Germany [4, 5]. Non-Germans (20–40 %, depending on year and list) were excluded. The lists also provided information on the professions, which showed that the condition for application of the Lincoln-Petersen-Chapman model was not strictly obeyed. The (limited) consequences hereof are investigated in Appendix 2. Details on calculations are given there as well. The estimates of the number of German X-ray users are shown in Table 5.

**Table 5:** Estimated number of German X-ray users (with standard deviation)

	1905	1906	1907	1908
From DRG-members (sd)^a^	810 (256)	769 (187)	990 (231)	1170 (275)
From DRG-congress visitors (sd)^a^	1022 (194)	627 (144)	838 (218)	1011 (261)

^a^ standard deviation reflects Chapman’s uncertainty only, total uncertainty larger (Appendix 2)

In 1905 the DRG held its first congress, which in comparison to congresses in the next years attracted many visitors, 512 versus 214, 249 and 350 in 1906, 1907 and 1908, respectively, with possibly persons interested in X-rays, but not involved in their use. This might explain the high congress based estimate of users for 1905, as this estimate will be too high by approximately the same percentage as the percentage of no-X-ray users in the congress list.

For the US we had earliest member lists of the American Roentgen Ray Society (ARRS) for 1902 and 1903 [3]. In the 1903 list, the year of first membership was indicated, so lists for 1900 and 1901 could be reconstructed, but notice that members who left the society again before 1903 are missed. Non-US citizens (1.4–3.5 %) were excluded. The results are presented in Table 6.

**Table 6:** Estimated number of US X-ray users (with standard deviation). Standard deviation reflects Chapman’s uncertainty only, total uncertainty is larger (Appendix 2)

	1900	1901	1902	1903
From ARRS-members (sd)	268 (58)	382 (78)	536 (89)	609 (94)

From the data collected for estimating population sizes, we can also calculate the fraction of X-ray users at that moment who were to be future victims (“prevalence”). For Germany this could be done separately for medical doctors and suppliers. Results from German member and congress lists were averaged for better statistics (Table 7).

**Table 7:** Fraction (%) of persons on member/congress list who are future victims

	ARRS 1900	ARRS 1901	ARRS 1902	ARRS 1903	DRG 1905	DRG 1906	DRG 1907	DRG 1908
All (md + sup + tech)	15.5	11.3	8.3	7.7	4.4	6.0	4.6	3.8
Medical doctors					4.4	4.9	3.6	2.8
Suppliers					5.6^a^	11.7	13.7	12.7

^a^ Not a single supplier-victim among the members in 1905, causing this low estimate

For the US there is a strong decrease with time; for Germany the decrease is less marked. With time, beginners were less prone to becoming a victim, lowering the percentage of all future victims within the group.

In Germany the fraction of future victims was considerably higher for suppliers than for medical doctors (two bottom rows in Table 7); taking the ratio of the summed rows shows that this risk was a factor 2.8 higher. This is in line with Hesse’s writing in 1911 about the incidence of skin malignancies, as he stated that “in the early years of the first decennium mostly engineers were affected, in recent years medical doctors” [25].

In estimating population sizes we talked about “X-ray users”, but is this correct? According to the Ehrenbuch nearly all victims had worked with X-rays; only for 22 martyrs from the total of 412 was the use of radium reported without mentioning the use of X-rays.

Finally, it is likely that parallel worlds of X-ray users have existed, for instance, of technically oriented persons who studied X-rays, of practitioners who used X-rays incidentally, but never associated with societies, and of workers in beauty parlours who applied X-rays for hair removal. These operators remained invisible in our analysis. How large (or small) these groups were we don’t know. Our results seem to apply to the medically oriented group of established X-ray users, for which radiology was an important aspect of their work. The suppliers of their equipment are clearly included.

## Risk to become a martyr

**Fig. 3:**
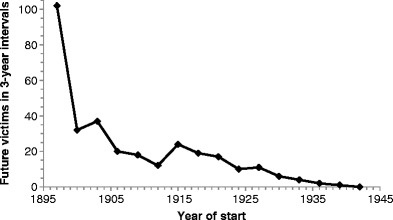
Number of future victims as a function of starting year of radiation work (n = 317); all causes of death. Victims summed over 3-year intervals to smooth the graph (e.g. 1897 represents the sum of 1896–1898)

Figure 3 shows the number of X-ray workers who started in a given year and who became a martyr. This incidence of future victims in each year is the product of the number of beginners and their risk. As argued in Appendix 3, the increase in X-ray users can probably, within broad limits, be considered linear, the annual accrual in workers thus as approximately constant. Consequently, Fig. 3, being the product of risk and a constant, should approximately reflect the relative change of risk over time. Figure 3 thus suggests an initially higher risk, followed at first by a sharp and later by a slower decrease of risk. During World War I (1914–1918) large numbers of X-ray systems were installed, frequently used by poorly trained personnel under primitive circumstances. Fluoroscopy was often used in preference to radiography to avoid both the delay associated with film processing and its financial cost. These facts might explain the small bump in the graph around this time. As discussed in Appendix 4, the causes of the overall decrease in risk were both of technical and behavioural nature.

A calculation of the absolute risk is also possible, as it is the ratio of the known incidence of future victims and the also known number of annually starting pioneers. The incidence of future victims is obtained from a graph similar to Fig. 3 for the country of interest, scaled up for victims with missing starting years (20 % for Germany, 29 % for the US). For Germany the incidence of future victims was 2.8 persons/year during 1905–1908, while the number of annually starting X-ray users was approximately 220. This latter value is the average of the slopes of the two linear fits to the 1906–1908 estimates of the size of the X-raying community, thus neglecting the 1905-outlier (Fig. A2 in Appendix 2). These values yield an absolute risk of 1.3 %.

**Fig. 4:**
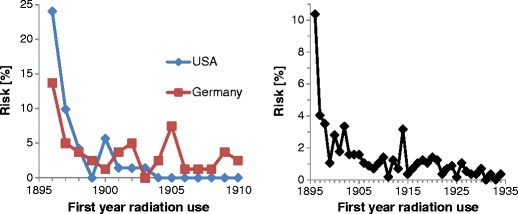
Approximate risk to die from a radiation induced disease as a function of starting year. Left, risk in Germany (n = 57) and US (n = 41). Right, all countries in the Ehrenbuch together (n = 317)

A similar analysis for the US for the years 1900–1903 gives a risk of 2.1 % (=100 × 2.5/118). The US result might indeed be expected to be slightly higher than the German value, as it comes from an earlier period, but considerable uncertainty in both numbers must be assumed (anyhow, the risks are small). The (“noisy”) risk curves for Germany and the US are presented to the left in Fig. 4. They show high (but somewhat speculative) initial risks of 13 % and 24 % in 1896, respectively. As a check on numerical errors, the member based percentages in the top row of Table 7 were recalculated using the Fig. 4 risks for Germany and the US, which gave the required agreement (between Fig. 4 and the top row of Table 7 exists 100 % dependence).

It is too tempting not to use the absolute risk estimates for Germany and the US to generate a curve giving roughly the risk to become a martyr for all persons in the Ehrenbuch. Normalizing the incidence curve for the victims of all nations to these known risks in both time intervals (1900–1903 for the US, 1905–1908 for Germany) leads to Fig. 4 (right). Please note its uncertainties due to potential differences between countries in improving safety, deviations from linearity of the growth of X-ray communities, and errors in our Chapman population estimates.

## X-ray-induced skin malignancies

In this section we will only deal with X-ray users who died from metastasized skin cancer, forming 71 % of all victims with known cause of death. Hematologic disorders are complicated and deserve a separate study; electrocution was addressed before [26]. We also excluded radium users, even if they had also applied X-rays, because their time characteristics were different and because the penetrating gamma rays and particle emissions of radium cause potentially different effects (both external and internal exposure occurred). Nearly 200 skin cancer victims could be included.

**Fig. 5:**
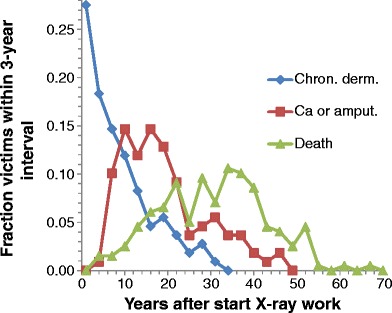
Time of incidence of some characteristic stages of fatal X-ray-induced skin harm: chronic dermatitis (n = 109), malignancy or finger loss (n = 109), and death (n = 198)

Figure 5 shows the incidence over time of some stages of late skin harm in X-ray victims. Finger loss was considered equivalent to the diagnosis of malignancy, as the latter often appeared to be implicit in cases of amputation. What is striking at first sight is the width of all distributions. Numerical data characterizing these distributions are given in Table 8.

**Table 8:** Time of incidence of various stages of X-ray damage after starting the use of X-rays; times in years

	Chronic dermatitis	Skin cancer or 1st loss finger	1st loss hand	1st loss arm	1st axillar involvement	Death
Average	8.49	19.53	22.35	21.66	25.25	30.44
sd_m^a^	0.72	1.03	2.06	1.63	1.69	0.86
sd^b^	7.53	10.80	9.90	10.81	12.18	12.13
Median	6	17	22	20.5	26	31
Sample size	109	110	23	44	52	198

^a^ sd_m is standard deviation of the mean; ^b^ standard deviation

Note that the sources in Table 8 differ (also to be seen from sample sizes). This probably explains arm loss preceding hand loss, but the difference is not statistically significant. Hood and Bunkis quote, in 1984 and 2004, respectively, a latent period for developing skin cancer of 25–30 years [27, 28], somewhat larger than our value for probably more severe cases. Hesse gave a thorough and often cited assessment in 1911 [25] more estimates to compare with (Table 9).

**Table 9:** Temporal characteristics of radiation induced afflictions according to Hesse. The range is given in square brackets; n gives Hesse’s sample size [25]

Start - Chronic dermatitis [years]	n	Start - Cancer [years]	n	Chronic dermatitis - Cancer [years]	n	Start - Death [years]	n
7½ [1–11]	28	9 [4–14]	37	4½ [1–11]	20	9½ [5–13]	10

Our results in Table 8 are all larger than Hesse’s values. It should be realized, however, that Hesse in 1911 had only 15 years of practice to look back on, so he will have missed many of the less severe and later cases. Moreover, treatment of chronic dermatitis may have become better in later years with less wait-and-see and improved surgery.

What can be said about the X-ray doses these victims incurred? Here follows a brief discussion; more information is presented in Appendix 4.

According to ICRP 118 [29], tissue reactions after a single exposure start at about 2 Gy with erythema, while at about 24 Gy ulceration is induced. At intermediate doses, loss of hair and dry and wet shedding of the skin occur. X-ray pioneers reported all these forms of acute damage, showing the range of doses they received in unfortunate single exposures. Long-term damage, to be distinguished from acute harm, is induced in 50 % of the irradiated persons by a single exposure of 17 Gy and by chronic exposure of 69 Gy (cumulative dose). Thus, skin cancer victims must generally have incurred doses of this order of magnitude, and often higher.

From the biographies it appears that several victims received rather massive doses in a relatively short time (in a single event or in weeks to months). These persons could develop severe chronic dermatitis after a short period. Generally the severe form proved unhealable and was only to be kept in control by surgery. Malignancy could evolve fast, and even if metastases were generally slow to develop, without adequate surgery, death by metastasized disease would follow. Unfortunately, the choice between preventing metastases and suffering invalidation by losing (part of) an upper extremity was a precarious one, as proven by the many names in the Ehrenbuch.

There was also another scenario. A worker could accumulate a slowly increasing dose without untoward signals. Several operators reported that they had believed themselves to be insensitive to X-rays, as no serious injuries were noticed, until (sometimes very much) later the body would respond with chronic dermatitis. It was quite possible that at that time the occupational exposure had long ago been reduced to a more acceptable level. The fate of many victims was thus often unknowingly determined early in their career, with no later possibility to halt the tissue reactions leading to future misery. Manifestation of the damage might be triggered by a small (e.g. blunt) trauma. Clearly, ending X-ray work gave little benefit in such cases, although rest or vacation was reported to (temporarily) improve conditions in some instances.

The widths of the distributions in Fig. 5 illustrate how different the dose related temporal development could be. The first victim (Clausen) died in 1900 [2]; he and persons similar to him make up the left parts of the distributions in Fig. 4. A person who lived into old age was Dessauer [2]; he died from metastases after more than 100 interventions, aged 81 years. The rightmost point in Fig. 5 at 67 years is his; interestingly, he started experimenting with X-rays at the age of 15.

## Concluding remarks

We hope to have contributed to the understanding of early radiology by giving answers to questions about the number of early X-ray users in a medical setting in two major countries, the pioneers’ risk to become a radiation victim, and the induction of fatal skin afflictions including their changes over time. Some background information on early (lack of) understanding of X-rays, experimental circumstances, operator behaviour, and the radiation doses incurred by the X-ray victims was provided to explain their fate.

Great accuracy is not claimed as many assumptions had to be made and no solid method to check the Chapman estimates was available. Moreover, some sources may not have been accurate, for instance, there is a natural uncertainty in the time chronic dermatitis or malignancy can be said to be present. In several cases we also found (usually minor) discrepancies between sources, and errors in interpreting qualitative time information may have been made. However, we think their effect on the main messages is small.

## Electronic supplementary material

Below is the link to the electronic supplementary material.

ESM 1 (PDF 253 kb)
