# Supplementary material for: Early X-ray workers: an effort to assess their numbers, risk, and most common (skin) affliction
Source: Insights Imaging. 2015 Dec 29;7(2):275–82. doi: 10.1007/s13244-015-0457-2 (PMC4805624; doi:10.1007/s13244-015-0457-2)
Supplement: Supplementary file 1 — (PDF 253 kb) [file 13244_2015_457_MOESM1_ESM.pdf]

Early X-ray workers: an effort to assess their numbers, risk and most common (skin) affliction

Appendix 1 Shortening of the lifespan of radiation victims

It is not trivial to obtain a good estimate of the shortening of the life of a radiation victim as one needs a suitable reference lifespan. At the time of interest, say 1850-1910, life-expectancy-at-birth is not a good indicator for the lifespan of any member of the working population, as it might be today. This is caused by the high childhood mortality, accounted for in life-expectancy-at-birth, but no longer relevant for any person who passed youth. To illustrate the effect, we calculated the average lifespan for the German victims in the Ehrenbuch using life-expectancy-at-birth data, finding a value as low as 41±3 years (n=64), much lower than the actual average age at death of 63 years in Table 4.

A better approach to calculate a reference lifespan is using actual time data of a victim, i.e. the age and the year-AD at which this future victim started working, in combination with age and year dependent life expectancy that gives the length of remaining life (Table A1). Effectively we then compare 'X-ray life' of a victim with the life of a double in the relevant phase of life only. In this approach the age distributions within the groups to be compared are automatically identical, the importance of which has been stressed by Seltser and Sartwell [30]. Suitable life expectancy data were found for France [31], Germany [32], the UK [33] and the USA [34,35]. These consisted of so-called cohort data for the civil population which distinguished between males and females, but not between social classes. The word 'cohort' indicates that the risk to die was taken as time (year AD) dependent.

Health is dependent on socio-economic circumstances and so is the lifespan. Our victims will generally have belonged to the class enjoying higher levels of longevity, thus our projections with general population data probably underestimate a victim's unimpeded lifespan. This possible underestimation of loss of years lived may partly be compensated by disease not directly related to radiation that shortened the lifespan of an already wasted victim.

Table A1 Projected, actual and shortening of lifespan of radiation victims

Country	n	Projected		Actual		Shortening[a]	
		Average	sd	Average	sd	Average	sd[b]
France	46	69.9	4.0	56.5	11.0	13.4	11.2
Germany	56	70.8	2.6	63.0	13.4	7.8	14.4
UK	39	69.9	2.3	60.1	12.0	9.8	11.9
USA	41	65.3	2.5	58.0	14.6	7.3	14.1
Combined	182	69.1	3.6	59.6	13.0	9.5	13.2

[a] Lifespan shortening in France is somewhat larger than in Germany and USA (both p=0.03)

[b] Standard deviation of the paired differences

In 43 of the 182 cases the actual lifespan exceeded the projected value.

In conclusion, the average lifespan of a radiation victim was shortened by about ten years compared to the lifespan of a double in the general population. With respect to a no radiation using peer this difference might be somewhat larger.

Appendix 2 Estimation of the number of early X-ray users in the US and Germany

A complication in our application of the Chapman model is that the individuals belong to different professional groups with potentially different risks and different likelihoods of being a member of a radiological society, or of being present at one of its meetings.

To investigate the consequences of this we will use the mathematically simpler Lincoln-Petersen formalism, which should be sufficiently representative for the similar Chapman modification. As indicated before, nearly all X-ray users fall into one of three professional categories, that of medical doctors (*md*), suppliers, (*sup*), or technicians (*tech*). We will consider the situation in which the suppliers and technicians have a risk and a representation within societies or congresses which differ from those of medical doctors. Suppose the risk to become a martyr at some time is for a medical doctor f, for a supplier αf and for a technician βf, respectively. Assume further that the probability that a doctor will be a society member or

visiting a congress is *F*, a supplier γF and a technician δF. The previously given equation, $N=(N_E/N_{SE})*N_{ST}$, can be transformed using

$$N_E = N_{Emd} + N_{Esup} + N_{Etech},$$

$$N_{SE} = F\left(N_{Emd} + \gamma N_{Esup} + \delta N_{Etech}\right) \; and$$

$$N_{ST} = \frac{F}{f}\left(N_{Emd} + \frac{\gamma}{\alpha}N_{Esup} + \frac{\delta}{\beta}N_{Etech}\right),$$

into

$$N_{LP} = \frac{1}{f}\left(\frac{N_{Emd} + N_{Esup} + N_{Etech}}{N_{Emd} + \gamma N_{Esup} + \delta N_{Etech}}\right)\left(N_{Emd} + \frac{\gamma}{\alpha}N_{Esup} + \frac{\delta}{\beta}N_{Etech}\right) \; (Eq. A1),$$

with N_{Ex}, $x = md$, *sup*, or *tech*, the number of victims from a certain country present in the Ehrenbuch and working at the time of the estimate. The correct expression for the population size is obviously the number of victims in each group divided by the group's risk summed

$$N_{true} = \frac{1}{f}\left(N_{Emd} + \frac{1}{\alpha}N_{Esup} + \frac{1}{\beta}N_{Etech}\right) \; (Eq. A2).$$

Notice that Eq. A1 and Eq. A2 give the same result if $\gamma = \delta = 1$, independent of the values of α and β. This of course reflects the basic condition for the validity of the model. But when γ and δ are not equal to 1, the outcome of Eq. C1 depends on all four parameters α, β, γ and δ. Equations A1 and A2 can be used to find the difference between the Lincoln-Petersen estimate and the true value for various combinations of α, β, γ and δ.

In the simulations we tried the following variations: $0.75 \leq \alpha \leq 3$, $0.75 \leq \beta \leq 1.5$, $0.6 \leq \gamma \leq 1.5$ and $0 \leq \delta \leq 1$ in a total of 400 (=4*4*5*5) combinations, as it is expected that this parameter space amply covers reality. N_{Emd}, N_{Esup} and N_{Etech} were taken from the Ehrenbuch for the country of interest. Notice that each combination of the parameters α, β, γ, δ, f and F corresponds to a N_{SE} and N_{ST} of a particular size and composition of medical doctors, suppliers and technicians which we could have encountered in our study. The simulation then shows the population size estimate and by how much it would have been wrong.

For Germany the ratio of the Lincoln-Petersen estimate and the true value ranged between 0.82 and 1.12. For the US the range was from 0.93 to 1.06. We saw these results as a justification of applying the Chapman model to the whole group of medical doctors, suppliers and technicians, as the magnitude of the potential error was considered acceptable in the light of all other uncertainties.

For Germany we do in fact have more information. We had found a *sup*/*md*-risk ratio (α) of 2.8. An estimate for γ could be obtained from separate Chapman group size estimates for medical doctors and suppliers, and the known number of (*md* and *sup*) members or congress visitors. This gave $\gamma = 0.75$ (note: for technicians the Chapman estimate was not possible due to lacking information). Technicians were virtually absent in the lists, only the congresses of 1907 and 1908 counted two and nine "Fräulein" visitors, respectively, who might well have been technicians. So δ will have been very small. Using α=2.8, γ =0.75, β=1 (assumption) and δ =0.05 (not critical), gave a ratio of 1.02. The same parameters gave for the US also a ratio of 1.02.

We will illustrate the calculation of the number of X-ray users for the US. The total number of (future) victims (N_E) living in each of the years 1900-1903 was estimated from biographical data (from the Ehrenbuch and other sources). For the US in 71% of the cases time data were available, and the so obtained (too low) estimate was corrected for missing data by scaling with (1/0.71), assuming the missing data had the same temporal distribution as the rest (may result in non-integers). The other two required numbers (N_{SE} and N_{ST}) were obtained by counting in member lists.

The Chapman equations are ('*var*' stands for variance):

$$N_{Chap} = \frac{(N_E + 1)(N_{ST} + 1)}{N_{SE} + 1} - 1,$$

$$var(N_{Chap}) = \frac{(N_E + 1)(N_{ST} + 1)(N_E - N_{SE})(N_{ST} - N_{SE})}{(N_{SE} + 1)^2(N_{SE} + 2)}.$$

The results are shown in Table A2 and Fig. A1; those for Germany in Fig. A2.

It is difficult to confirm our estimates, but a rough consistency check on the number of X-ray workers in the US in 1903 is possible. Pitkin stated in that year "about one-third of the prominent operators and instrument dealers have hands which have been more or less severely

injured” [36], and Hesse in 1911 reported that about 25% of those who developed a malignancy died [25]. Assuming that 100% of the ‘röntgen-hands’ developed cancer in the long run, the number of persons to die from the X-ray users working in 1903 is estimated as 609* ⅓ *1.0 *0.25 =51. The number of future victims actually at work in 1903 amounted to 48. The good agreement must be partly fortuitous, as the uncertainties are considerable. For instance, the ‘one third’ of Pitkin is probably an educated guess, and the assumed fractions of 100% and 25% may be off, the first possibly being too high, the second too low, as Hesse had only a short period for observation, or too high if later improvements in surgical interventions prevailed.

Table A2 Chapman’s estimate of number of X-ray users in the United States

	1900	1901	1902	1903
Living US martyrs in ARRS, N_{SE}	11	13	18	20
All living US martyrs, N_E	44	45.3	46.7	48
Number of US ARRS members, N_{ST}	71	115	213	260
Total X-ray workers (sd)[a]	268 (58)	382 (78)	536 (89)	609 (94)

[a] standard deviation reflects Chapman’s uncertainty only, total uncertainty larger

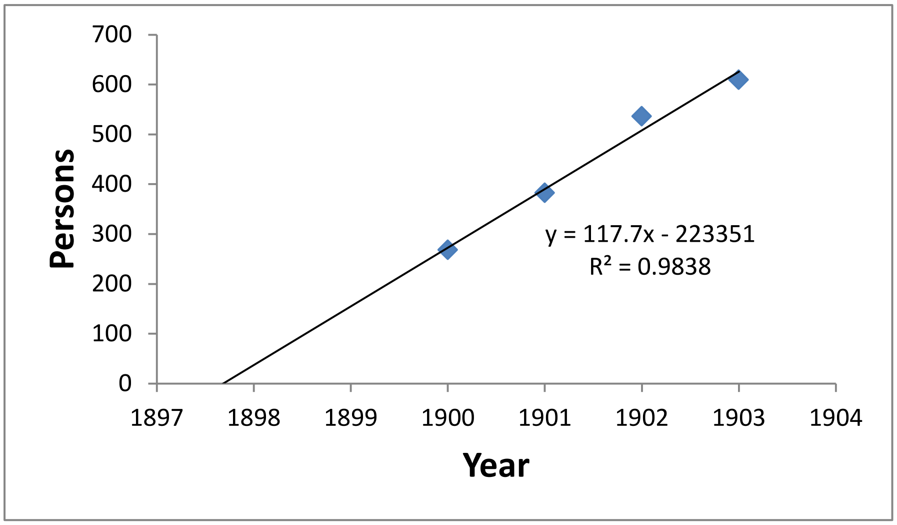

Fig. A1 Estimated number of US X-ray users

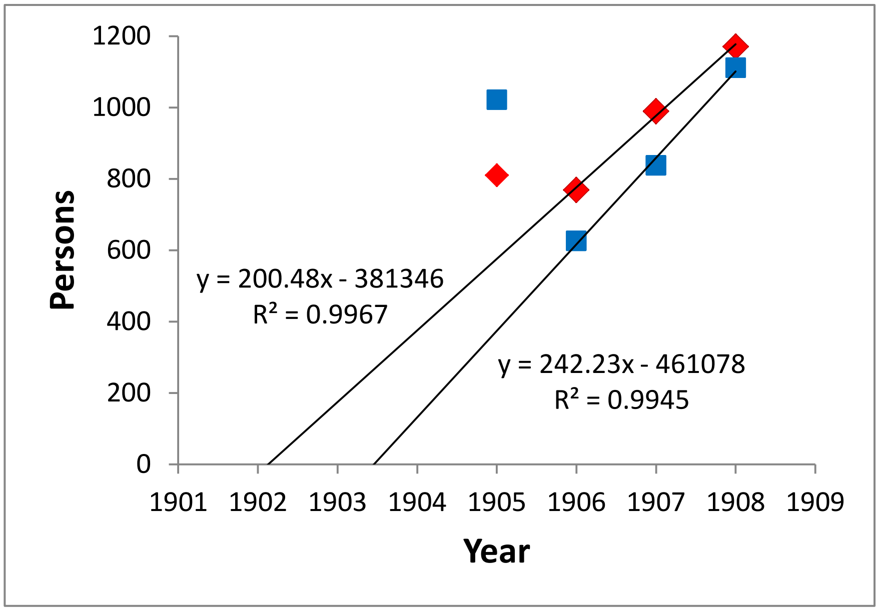

Fig. A2 Number of German X-ray users estimated from DRG members (red) and congress visitors (blue). Linear fits to 1906-1908 data only

Another (order-of-magnitude) check might consist of extrapolating the number of members of a radiological society in a certain area, where one expects the level of association with a radiological society to be high, to the whole country. We assumed the cities of Berlin and New York to be such areas. The results are shown in Table A3.

Table A3 Extrapolation of number of society members in reference area to whole country

	US - ARRS		Germany - DRG	
Year	1902	1903	1905	1908
Members in reference region	16	19	34	62
Members in country	221	269	126	296
Fraction region/country [%]	7.2	7.1	27	21
Inhabitants reference region	3.703E6	3.836E6	2.040E6	2.057E6
Inhabitants country	79.415E5	81.017E6	60.200E6	62.720E6
Fraction region/country [%]	4.7	4.7	3.4	3.3
Extrapolated nr of members	343	401	1003	1890
Chapman estimate X-ray users	536	609	810	1170

When not all X-ray users in the reference region were a society member the extrapolation will be too low as an estimate of the number of X-ray users in the country; on the other hand, a higher density of users in the reference area than the country average would lead to an overestimation. Especially in Berlin the density of society members seems to be very high (compare the two ‘Fraction’ rows). Considering the possible (but unknown) magnitudes of these counteracting effects, the extrapolations look compatible with the Chapman estimates, but no more can be said.

Appendix 3   Interpretation of the graph giving the number of future X-ray victims as a function of their starting year (Fig. 3 in main article)

Data from the Netherlands (Fig. A3) show a nearly linear increase in number of X-ray users. The top curve in Fig. A3 is the cumulative graph of persons who came for the first time to a meeting of the Dutch radiological society NVvER. It neglects the persons who died or stopped their work for another reason, on the other hand it misses users who never went to meetings. The bottom curve gives certainly an underestimation of the growth of X-ray users, as many of what we defined as ‘leavers’ probably only stopped visiting meetings while continuing their practice.

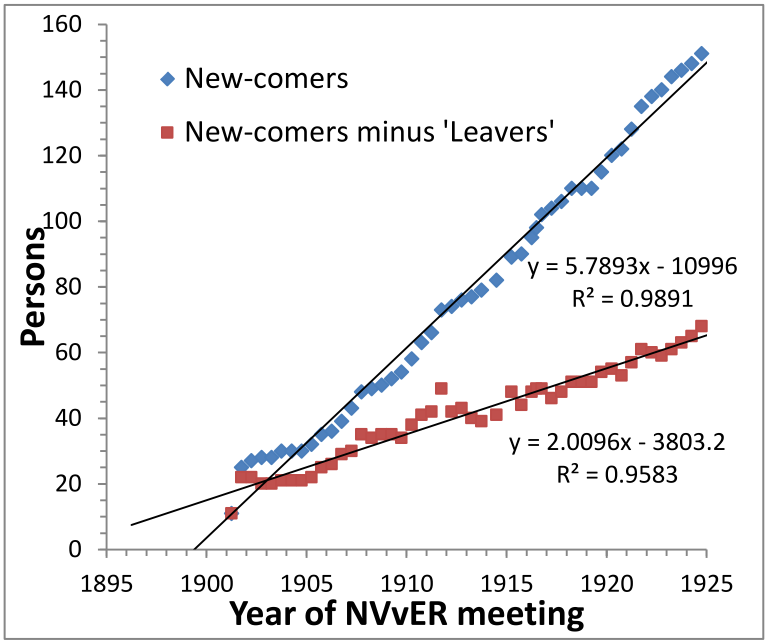

Fig. A3 Top curve shows running integral of all new-comers at Dutch NVvER meetings, the bottom curve the difference between new-comers and ‘leavers’, where a ‘leaver’ is defined as a person who is not to appear again at a meeting

Data for the US and Germany also give a linear relationship between the number of members and time (Figs. A4 and A5).

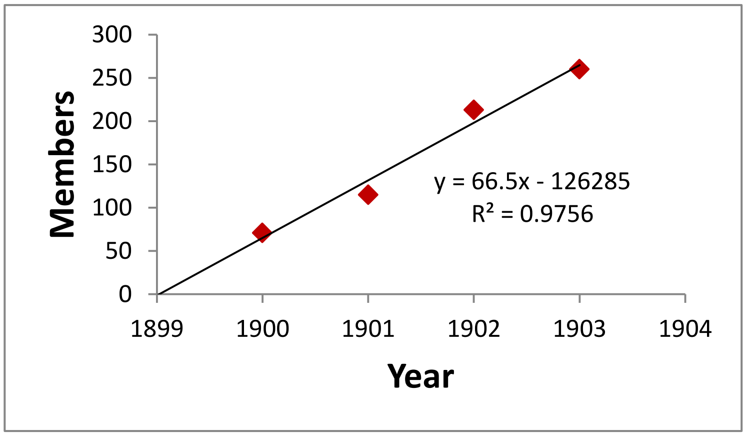

Fig. A4 Number of ARRS members from 1902 and 1903 member lists. Numbers for 1900 and 1901 were taken from the 1903 list as this gave the year of entering the society. Non-US members excluded

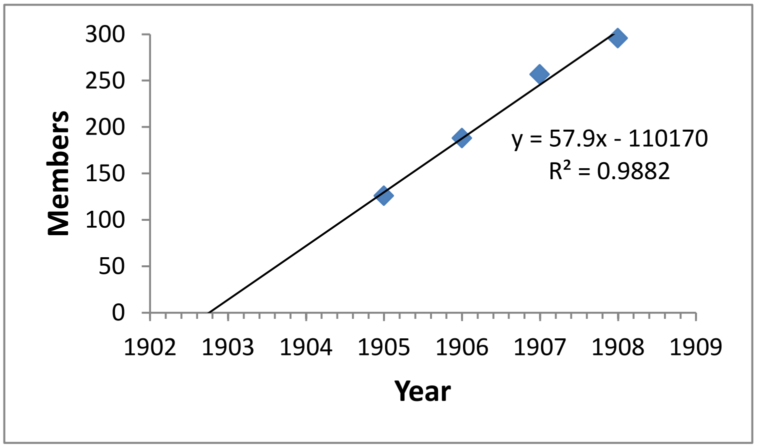

Fig. A5 Number of DRG members from 1905-1908 member lists. Non-German members excluded

Unfortunately, none of the three graphs (Figs. A3-A5) includes data from before 1900. Notice, however, that all linear fits cross zero after 1895, except the (probably unrealistic) red curve in Fig A3. Can we learn something from this for the period before 1900?

Consider three hypothetic scenarios for the growth of the number of X-ray users over time, L, L+ and L− in Fig. A6 (left). In blue (L) a linearly in size increasing group (with 100 persons/year) is shown, in red (L+) a scenario with a large initial group of starters of 300 persons, followed by the same linear increase of users as in L. Scenario L− (green) represents an annual increase that is initially lower than the 100 persons per year that is reached in 1900 and then maintained. Note that a linear fit to group size curve L+ would cross zero at a time before 1895, the year of discovery of X-rays, that to L− after 1895. This latter scenario (L−) appears to be corroborated by Figs. A2-A5. For simplicity (and being conservative in risk estimation) we assume the same constant linear increase in x-ray users also in the very first years after 1895, realizing that the annual growth of users may be overestimated (or just be more uncertain) in those years.

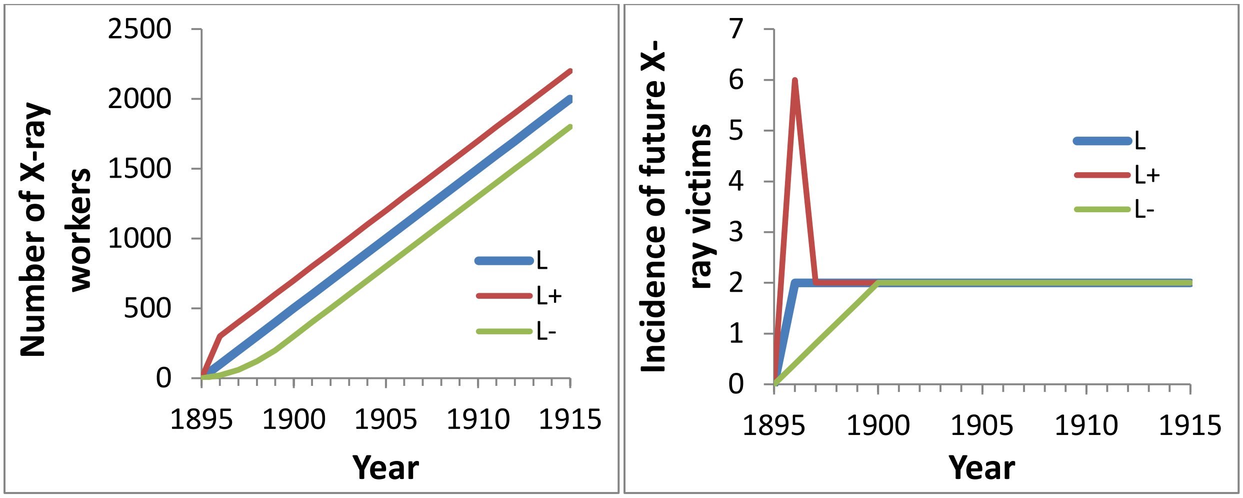

Fig. A6 To the left the increase of X-rays users for three hypothetic scenarios: linear increase from start (blue, L), large starting group followed by same linear increase (red, L+) and an increasing number of annual starters until also 100/year is reached (green, L−). To the right the incidence of future martyrdom assuming a constant risk of 2%

It may be interesting to see how a graph like Fig. 3 would have looked for the three scenarios had the risk to become a martyr been constant over the years. Assuming an arbitrary risk of 2%, the equivalents of Fig. 3 are shown in Fig. A6 (right). The red peak (of 6, i.e. 2% of 300) corresponds to victims from the large initial group in the scenario L+, the rising green part to victims from the group with the initially lower annual growth (scenario L−). The constant part

is for all scenarios the (identical) derivative of the parallel lines in Fig. A6 (left) times the risk to become a martyr (100*0.02=2). Without the arguments given above, scenario L+ might have come to mind when noting the correspondence between the initial peak in Fig. 3 and that in Fig. A6 (right, red). Rejecting scenario L+, the sharp drop in Fig. 3 can only be explained if the risk decreased over time. Independent testimony for an initially very fast reduction of risk, as Figs. 3 and 4 in the main article suggest, comes from data on burns in patients and operators collected by Codman (Fig. A7). In his 1902 article he writes “The main reasons for such a decrease have been the bitter teachings of experience and the fact that the introduction of better apparatus has done away with long exposures and the close approximation of the tube.”

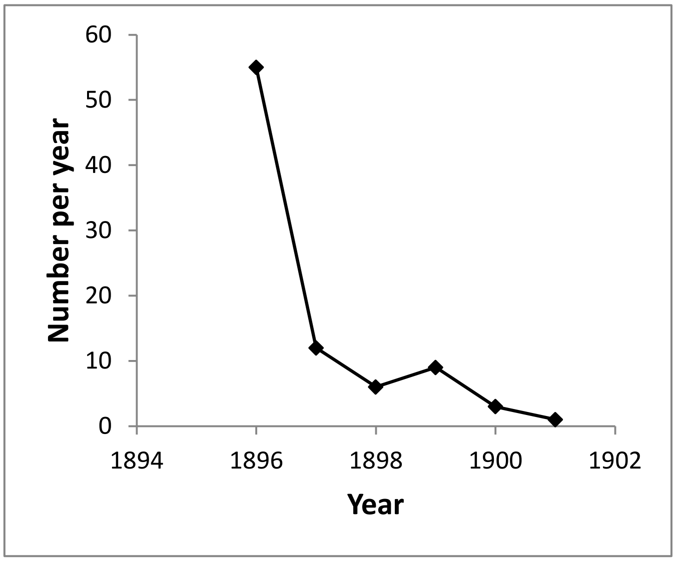

Fig. A7 Cases of X- ray burns in patients and operators with known date of occurrence (n=86) collected by Codman [37]

If our interpretation of Fig. 3 is correct, it seems that in 1896 or 1897 a large part of the critical (high) exposure must have taken place in a rather short time immediately after starting X-ray work, because if the initial risk had stayed high for several more years, more persons who started in 1898 and 1899 would have become a victim. In later years, say after 1900, on the basis of Fig. 3 little can be said about the time that must have passed before critical harm was done. From the biographies it appeared that in many cases the exposure could have lasted many years, but with a view to the gradual improvement of shielding it is likely that the first years contributed most to the cumulative dose.

Appendix 4 On the exposure of early X-ray workers

To obtain an impression of the dose the X-ray victims received, it is worthwhile to look at ICRP 118, 'Early and Late Effects of Radiation in Normal Tissues and Organs – Threshold Doses for Tissue Reactions in a Radiation Protection Context' [29].

Table 2.2 of ICRP 118 gives threshold doses, i.e. doses for 1% incidence, for single exposures. The skin's response starts at about 2 Gy with erythema. Epilation occurs from about 3 Gy (temporary) to 7 Gy (permanent), dry shedding of the skin at 14 Gy, wet at 18 Gy, and ulceration at about 24 Gy. Such reactions are seen after hours to several weeks. Recovery depends on the dose and the size of the exposed area, and can last for months or even more than a year. Many of the earliest pioneers suffered such acute X-ray 'burns' in all these degrees of seriousness, for instance in self-experiments and demonstrations [37,38]. Very severe burns left the skin more vulnerable to further exposure. A slight complication here is that the unfiltered X-rays used by the pioneers were very soft so that their penetration was low and the dose in the skin possibly not uniform. So the surface dose may have been even higher than the ICRP dose for the same effect. Today noticeable acute tissue reactions in radiology are limited to patients undergoing very extended or suboptimally performed interventional procedures.

Of more importance in the present context, however, are the long term effects that usually take (much) more than a year to become manifest and which may include skin cancer. Again according to ICRP 118, persons exposed to an acute dose of 10.5 Gy have a risk of 1% to suffer from skin atrophy, after a dose of 17 Gy the risk has risen to 50%. Thus a single severe radiation accident can already have fatal consequences in the long term by (deterministic) tissue reactions. Corresponding doses by protracted exposure are 40 Gy (1%) and 69 Gy (50%). We assume that these latter ICRP values, determined from radiotherapy treatments of 30 fractions, are applicable to chronic exposure as might have existed in the case of the pioneers. Atrophy may be accompanied or followed, especially at higher doses, by various other distressing skin conditions, often called 'chronic dermatitis' [39]. The hand showing chronic dermatitis is known as a 'röntgen hand'. The ICRP values show how high (40 Gy-70 Gy and possibly higher) the skin dose of many (chronically) exposed X-ray victims must have been. Higher doses led to more serious afflictions and to a faster appearance [40]. Skin irritating chemicals used for developing photographic plates or films may have worsened damage caused by radiation. Effects

on deeper lying organs were much less frequently noticed, among others because the X-rays had low penetrating power.

ICRP 118 doesn't specify the full spectrum of late effects, probably because they no longer occur in a radiation protection context. We therefore cite from Cole's description of the more severe forms of chronic radiodermatitis [41]. Pigmentation ('tanning') is often seen as a first signal of exposure, already after low doses. After higher doses, however, pigmentation may completely and permanently disappear, as may the hair and dermal appendages like sebaceous and sweat glands. Often telangiectasia develops between a few weeks and about a year but it may progress for years. Injuries to the nails like longitudinal ridges and brittleness, or (often temporary) loss of nails are seen. Keratoses, raghades, callous patches and ulcers may form. Excruciating and untreatable pain is another debilitating consequence. Chronic dermatitis that remains unhealed is believed always to lead to cancer [27,42]. An impressive description of his experience with X-rays is given by (the later martyr) Pitkin in a poetic but in the end grim story [36]. About pain he writes: "For a description of the pain and suffering, hyperaesthesia and paraesthesia, no language, sacred or profane, is adequate."

In order to understand how early radiologists incurred the above mentioned skin damaging doses of X-rays one should look at 1.) their knowledge, 2.) their experimental circumstances and 3.) their behaviour. Several books provide information on these topics [6-15], but also two articles by Ratkóczy should be mentioned [43,44].

1.) Scientific knowledge of X-rays hardly increased for a long time after Röntgen's original publication of December 1895. Moreover, the available knowledge was not helpful in understanding the X-rays' effect on tissues [19]. Notwithstanding this limitation a lot of practical experience was acquired. 'Burns' (acute dermatitis) were observed within months in 1896 [45]. Severe cases, even with necrosis (or gangrene), followed in 1897 [38]. By 1902 it was also known that cancer could be induced [46]. These observations did not directly lead to appropriate measures to limit exposure. One important reason may have been the disagreement on the cause of the biological effects. The majority of users believed for quite some time, at least to after 1900, that electrical effects originating from the electrically activated tube and wires were responsible for the harm (an excellent discussion is given by Serwer [19], Chapter 2). This believe led to placing earthed metal foils between tube and user or patient. Although the metal

attenuated and filtered the X-rays to some extent, it gave no adequate protection. Several other causes were considered as well, idiosyncrasy was believed to play an important role as several operators had never contracted burns. Static high voltage generators were first (end1896) claimed not to 'burn' [47], later for several years believed to be more safe than induction coils [38]. These false beliefs caused unnecessary exposure. Interestingly, even after conclusive proof had been given that X-rays were the causing agent of the damage, the radiological community did not accept this immediately, as it was perceived as against longstanding experience [19]. All this probably delayed the development of protection by shielding from X-rays by a heavy material like lead, which was to become a key factor for more safety.

2.) Initially in 1896 the X-ray tube was completely bare, radiating in all directions, although it was later known that behind the anti-cathode the level of radiation was lower [38]. The primary X-ray beam produced a lot of secondary radiation by scattering from surrounding walls and other objects, which also exposed the photographic plate, fluoroscope, operator and patient. For improvement of image quality soon a diaphragm was introduced to limit the contribution of secondary radiation to the image. This also provided some protection for operator and patient. The introduction of the compression diaphragm (1903) still further reduced scatter, giving better image quality and a little more protection. Considerable reduction of the skin dose was achieved by using X-ray beam filters, e.g. of leather [48] and later aluminium [49]. To give an example, an Al-filter of 0.5 mm thickness reduces the air kerma of a 50 kV X-ray beam coming from a tungsten anti-cathode, with a 45 degree angle, only filtered by 1 mm glass of the tube wall and at 20 cm distance, from 2.67 mGy/mAs to 1.33 mGy/mAs, thus by a factor of 2.0. For 2 mm glass this reduction is 1.5 (=1.13/0.77) (estimates made with PCXMC, STUK-Radiation and Nuclear Safety Authority, Helsinki, Finland). The anode material at the time was platinum, with atomic number Z=78, while our PCXMC-estimates are for tungsten (Z=74). The X-ray output scales approximately with Z, thus was slightly higher than our estimates above. The filtering turned out also to improve image quality.

Rollins, a very prudent and ingenious dentist, started from 1898 onwards to place the tube in a box covered with white lead paint (white lead was an isolator, a metal, e.g. Pb, could not be brought close to the tube as it might facilitate discharges between anode and cathode) [50,51]. Unfortunately, Rollins' valuable work was neglected, even when his brother in law Williams, a respected radiologist and writer of a textbook, used Rollins' innovations in his practice and

showed them in his textbook of 1901. Later, after about 1902, bowls covered with lead sheet came into use. Still later bowls of lead-glass, in which the tube could rest, attenuated the X-rays in all directions except in the direction the beam was going to be used (there was a hole), and in the direction of the ceiling. This large opening at the top allowed positioning of the tube in the bowl. The transparent lead-glass bowl had the advantage that it facilitated observation of the gas discharge, necessary as a first check of the tube's functioning. A later alternative for the bowl was an X-ray tube made of leaded glass, except for a small window of normal glass for the exit of the X-rays. Later lead sheet was used more abundantly in the form of screens, between patient and observer, at the sides of the patient forming a box intercepting secondary rays, in movable shields and in the walls of cubicles for observers [44]. Lead foil or leaded rubber was used to shield the patient outside the X-ray field of view. The fluoroscope, at least the carrier of the fluorescing salt, was made of leaded glass, after less effective thicker but normal glass had been used. Additionally, the fluoroscope got lead extensions protecting the hand holding it. Lead gloves were introduced, as well as lead aprons. Convenient X-ray stands offering more protection were commercially taken into production (e.g. according to Béclère and Wenckebach, in about 1908 and 1910, respectively). Nevertheless, manufacturers were criticized for not being more pro-active in the field of safety [6]. After 1913 the high-vacuum hot-cathode tube (developed by Coolidge at General Electric) slowly replaced the classical gas tube. The new tube no longer needed visual observation as it was stable and very reliable. In 1928 the first fully self-shielded X-ray tube, the Metallix from Philips, came onto the market. At least after 1903 X-ray users could have had working conditions which no longer led to severe tissue reactions, as sufficient knowledge was available as were the technical means.

What can be said about doses based on their apparatus? We have seen above that at 50 kV the air kerma at 20 cm distance from an X-ray tube without additional filter could be as high as 2.7 mGy/mAs (1 mm glass wall). A tube current of 1 mA, corresponding to 50 Watt thermal dissipation, may have been used around 1900. A skin dose of 1 Gy would then be incurred in about 6 minutes. Playing around with an obstinate system may easily have led to doses in the 'burn' region, i.e. > 2 Gy, and far above if sufficiently procedures were performed. A lot has changed since these early days. Today the average radiation exposure in a radiology department may even be lower than in the workers' home [52].

3.) Because the source of the X-rays, the gas tube, was notoriously unstable, X-ray systems in 1896 must have required endless experiments to establish conditions that gave useable X-rays. As no harm was expected from exposure to X-rays, protection was not an issue. The worker would often stay close to the functioning X-ray system as can be seen on many early photographs. Burns were initially considered as no more dangerous than ordinary burns by heat or the sun. To gauge the quality of the radiation coming from the tube, the operator would use his hand as test object in front of the fluoroscope and close to the tube, while the other (also bare) hand held the fluoroscope (this was a second tube test, next to visual observation of the gas discharge). The whole body was in the primary beam. Fluoroscopy of patients was also a high exposure activity, as the operator was standing in the rays transmitted through the patient and in the scattered radiation coming from everywhere. And at moments where he was not in the patient's shadow he was exposed to the full beam. But in a discussion in 1903 Kassabian remarked that the number of dangerous fluoroscopies had decreased in comparison with the first years [36]. Assisting patients (e.g. children) during the making of radiographs, or holding the photographic plate, were other risky endeavours. Innumerous demonstrations with X-rays formed still another occasion for high doses because often the operator's body was used as test object. This could be for visitors at fairs or in shops, but also for clients in the case of manufacturers and salesmen of X-ray systems. Also in the production process of X-ray tubes the exposure could be very high, as there was a lengthy evacuation in an oven during which the functioning tube had to be observed. The heat served to drive residual gases out of the glass wall and metal parts. Some improvement in the stability of the gas tube was obtained by various forms of automatic vacuum regulation (1896 and later). In 1904 a phantom ('osteoscope') that could be used as a substitute for a hand in gauging radiation quality was produced by Max Kohl from Chemnitz (Germany) [53].

The reasons X-rays after 1903 still caused skin cancer fatalities (about 37% of all skin cancer deaths) are probably multiple. In arbitrary order: lack of education, economic reasons as both safety bringing hardware and a lower efficiency by prudent behaviour had its costs, falling into or continuing unsafe practices as radiation was not felt, ordinary slackness, large workload, extensive fluoroscopy, extreme professional zeal. The latter is a recurring characteristic in the

(often somewhat hagiographic) biographies. Although the number of X-ray victims is large, fortunately it involved only a relatively small fraction of all workers.

References (continued from main article)

30. Seltser R, Sartwell PE (1958) Ionizing radiation and longevity of physicians. J Am Med Assoc 166:585-7
31. The Human Mortality Database. France, Civilian Population, Cohort Data 1816-1922. Life tables, Females and Males, 1x1. Available via http://www.mortality.org/cgi-bin/hmd/country.php?cntr=FRACNP&level=1. Accessed on 14 November 2015
32. Statistisches Bundesamt, Destatis, Generationensterbetafeln 1896-2009. Trend V1. Männliche und weibliche Jahrgänge für das frühere Bundesgebiet. Available via https://www.destatis.de/DE/Publikationen/Thematisch/Bevoelkerung/Bevoelkerungsbewegung/Generationssterbetafeln5126101119004.pdf?__blob=publicationFile. Accessed on 15 November 2015
33. The Human Mortality Database. U.K., England & Wales, Civilian Population, Cohort Data 1841-1922. Life tables, Females and Males. Available via http://www.mortality.org/cgi-bin/hmd/country.php?cntr=GBRCENW&level=1. Accessed on 14 November 2015
34. National Vital Statistics Report, Volume 63, Number 7. Table 21. Life expectancy by age, race, and sex: Death registration States, 1900-1902 to 1919-21, and United States, 1929-31 to 2010. White male and female. Available at http://www.cdc.gov/nchs/data/nvsr/nvsr63/nvsr63_07.pdf. Accessed on 16 November 2015.
35. Infoplease. Life expectancy by age, 1850-2011. White males and females. Available via http://www.infoplease.com/ipa/A0005140.html. Accessed on 15 November
36. Pitkin JT (1903) Danger of the X-ray operation. Trans Am Roentgen Ray Soc 232-259
37. Codman EA (1902) A study of the cases of accidental X-ray burns hitherto recorded. Philadelphia Medical Journal 9:438-442
38. Kienböck R (1900) Ueber die Einwirkung des Röntgen-Lichtes auf die Haut. Wiener klinische Wochenschrift 13:1153 -1166
39. Unna, PG (1904/5) Die chronische Röntgendermatitis der Radiologen. Fortschr a d Geb d Röntgenstrahlen 8:67-91

40. Saunders TS, Montgomery H (1938) Chronic roentgen and radium dermatitis. An analysis of 259 cases. J Am Med Assoc 110:23-28
41. Cole H.N (1923) Röntgen-ray dermatoses as seen in the professional man. J Am Med Assoc 84:865-874
42. Porter CA (1908) The pathology and surgical treatment of chronic X-ray dermatitis. Transactions of the American Roentgen Ray Society. Ninth annual meeting, New York City, NY, December 28-30, 101-170
43. Ratkóczy N (1971) Geschichtliches über Strahlenschädigung und Strahlenschutz. Teil I. Strahlentherapie 141:311-320
44. Ratkóczy N (1971) Geschichtliches über Strahlenschädigung und Strahlenschutz. Teil II. Strahlentherapie 141:425-438
45. Marcuse W (1896) Nachtrag zu dem Fall van Dermatitis und Alopecie nach Durchleuchtungsversuchen mit Röntgenstrahlen. Deutsch med Wochenschr 22:681-682
46. Frieben A (1902) Demonstration eines Cancroids des rechten Handrückens, das sich nach langdauernder Einwirkung von Roentenstrahlen enwickelt hatte. Fortschr a d Geb d Roentgenstr 6:106-111
47. Frei GA (1896) X-rays harmless with the static machine. Electrical Engineer 22:651
48. Pfahler GE (1906) A roentgen filter and a universal diaphragm and protecting screen. Trans Amer Roentgen Ray Soc 217-224
49. Phillips CES (1907) The standardisation of radiations. Am Quarterly of Roentgenology 1:1-5
50. Rollins WH (1904) Notes on X-light, Note 36. Boston, p 42
51. Williams FH (1901) The roentgen rays in medicine and surgery., MacMillan, New York
52. Kemerink GJ, Frantzen MJ, de Jong P, Wildberger JE (2011) Less radiation in a radiology department than at home. Insights Imaging 2:275-80
53. Beck C (1904) Röntgen ray diagnosis and therapy. Appleton & Lange, New York, p 25
